# *DEK::AFF2* Fusion‐Associated Sinonasal Carcinoma With Intracranial and Orbital Involvement

**DOI:** 10.1155/crip/7248935

**Published:** 2026-09-15

**Authors:** Katelyn Elizabeth Golden, Josh Barlow, Hong Yu, David J. Hernandez, Fouad El-Dana, Neda Zarrin-Khameh, Songlin Zhang, Li Liang

**Affiliations:** ^1^ Department of Pathology and Immunology, Baylor College of Medicine, Houston, Texas, USA, bcm.edu; ^2^ School of Medicine, Baylor College of Medicine, Houston, Texas, USA, bcm.edu; ^3^ Department of Otolaryngology, Baylor College of Medicine, Houston, Texas, USA, bcm.edu; ^4^ Division of Genomic Medicine, Texas Children′s Hospital, Houston, Texas, USA, texaschildrens.org; ^5^ Department of Pathology and Laboratory Medicine, Ben Taub Hospital, Harris Health System, Houston, Texas, USA, lhsc.on.ca

## Abstract

Sinonasal carcinomas encompass a molecularly heterogeneous group of malignancies. *DEK::AFF2* fusion‐associated carcinoma is a recently recognized subtype of nonkeratinizing squamous cell carcinoma characterized by deceptively bland morphology yet clinically aggressive behavior. We report the case of a 67‐year‐old woman who presented with a rapidly enlarging sinonasal mass with extensive local invasion involving both the intracranial compartment and orbit. Histologically, the tumor was composed of cytologically bland cells with monotonous nuclei and exhibited a combination of exophytic and endophytic growth patterns that closely mimicked a sinonasal papilloma. Although the tumor shows predominantly squamous differentiation, focal extracellular mucin was also present. The brain‐invasive foci demonstrated tumor cells in sheets and nests floating within pools of mucin, a pattern focally reminiscent of mucinous adenocarcinoma. RNA sequencing identified a *DEK::AFF2* fusion with breakpoints at *DEK* exon 7 and *AFF2* exon 6, confirming the diagnosis. The tumor demonstrated positive PD‐L1 expression with a combined positive score (CPS) of 40 and a low tumor mutational burden (TMB) of 0.5 mutations/Mb. This case highlights the diagnostic challenges posed by *DEK::AFF2* fusion‐associated carcinoma, underscores the importance of molecular profiling for accurate classification, and demonstrates the aggressive clinical behavior of this rare entity.

## 1. Introduction

The sinonasal tract, including the nasal cavity and paranasal sinuses, is a complex anatomic region that gives rise to a diverse spectrum of neoplasms and presents one of the most challenging differential diagnoses in surgical pathology [1]. Recent molecular studies have demonstrated that sinonasal carcinomas represent a biologically heterogeneous group of neoplasms. Distinct molecular subsets, including tumors characterized by *IDH2* mutation, *NUTM1* rearrangement, SWI/SNF complex deficiency, and human papillomavirus (HPV) association, have refined their classification and improved understanding of tumorigenesis [1].

*DEK::AFF2* fusion‐associated carcinoma is a recently recognized subtype of nonkeratinizing squamous cell carcinoma characterized by recurrent *DEK::AFF2* fusions, which are rare rearrangements involving the chromatin‐associated gene *DEK* and the transcriptional regulator *AFF2*. These tumors most commonly arise in the nasal cavity, although cases have also been reported in the middle ear/temporal bone, nasopharynx, and orbit [2–7]. To our knowledge, intracranial and orbital involvement occurring concurrently in *DEK::AFF2* carcinoma has not been previously described in the literature.

## 2. Case Presentation

**Figure 1:**
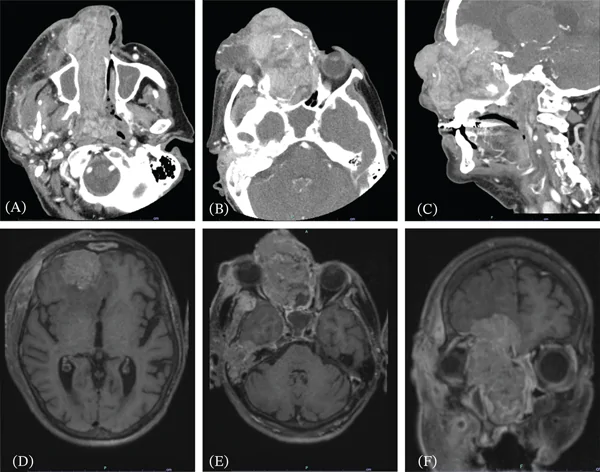
CT (A–C) and MRI scans (D–F) demonstrate a large nasal mass with intracranial and orbital involvement.

A 67‐year‐old woman with a history of smoking and colon cancer presented with a painful right facial mass. She reported having first noticed the mass approximately 1 year earlier, with rapid progression over the preceding 4–5 months. Imaging demonstrated a 7.7 × 7.0 × 3.6 cm heterogeneous mass centered in the right nasal cavity, with marked local invasion and bony remodeling (Figure 1). Superiorly, the mass extended beyond the skull base into the right frontal bone, frontal sinus and superior orbital wall, with extension into the anterior cranial fossa associated with vasogenic edema and approximately 9 mm leftward displacement of the falx cerebri. Posteriorly, it extended into the nasopharynx. Medially, there was extensive resorption of the nasal cavity and ethmoid air cells. Laterally, the mass invaded the right medial rectus muscle, causing marked lateral displacement of the right globe. Associated soft tissue swelling was present in the right periorbital region.

**Figure 2:**
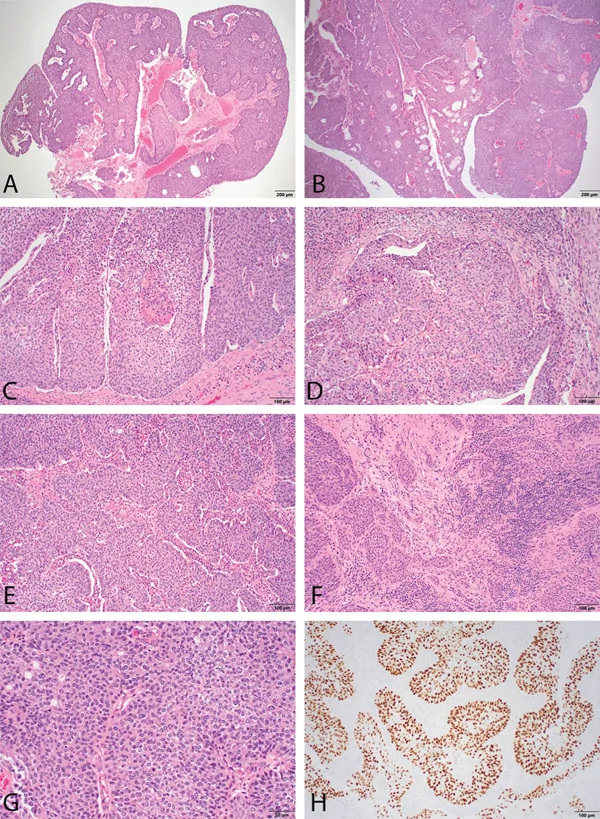
(A, B) Low‐power photomicrographs show a carcinoma with both exophytic and endophytic (inverted) growth patterns, mimicking a sinonasal papilloma. However, the overall architecture is more complex and confluent, (C–E) featuring anastomosing papillary and trabecular patterns. (C) Acantholytic changes and foci of central comedonecrosis are present. (F) Rare areas demonstrate an infiltrative pattern with irregular tumor nests. (G) On high‐power examination, the tumor cells are monotonous with eosinophilic to clear cytoplasm, round‐to‐oval nuclei and fine chromatin, characteristic of the deceptively bland morphology associated with *DEK::AFF2* squamous cell carcinoma. The tumor is diffusely and strongly positive for p40 (H), confirming squamous differentiation. (A–B: 40 ×; C–F, H: 100 ×; G: 200 ×).

**Figure 3:**
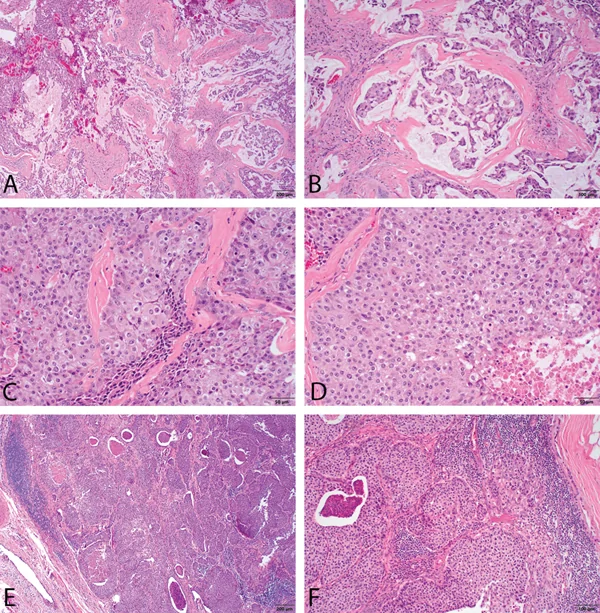
(A–D) Within brain‐invasive foci, the tumor cells form sheets and nests floating in mucinous pools, focally mimicking mucinous adenocarcinoma. (E, F) Sections from the lymph node metastasis demonstrate replacement of the nodal architecture by nests and sheets of tumor cells exhibiting cytologic features identical to those of the primary sinonasal tumor. The findings underscore the aggressive biologic potential of *DEK::AFF2* fusion‐associated carcinoma. (A, E: 40 ×; B, F: 100 ×; C, D: 200 ×).

The initial biopsy was interpreted as dysplastic squamous epithelium because of limited sampling. A repeat biopsy was subsequently performed. Histologic examination showed a tumor composed of cytologically bland cells with monotonous nuclei, exhibiting a combination of exophytic and endophytic growth patterns that closely mimicked a sinonasal papilloma (Figure 2). The tumor was diffusely positive for p40, p63, and cytokeratin 5/6. The tumor was negative for p16 overexpression and NUT, with retained nuclear expression of INI1 and BRG1. EBER in situ hybridization was negative, and p53 immunostaining demonstrated a wild‐type pattern. Although the tumor shows predominantly squamous differentiation, focal extracellular mucin was also present. Within the intracranial component, the tumor formed sheets and nests with abundant extracellular mucin, focally mimicking mucinous adenocarcinoma (Figure 3).

**Figure 4:**
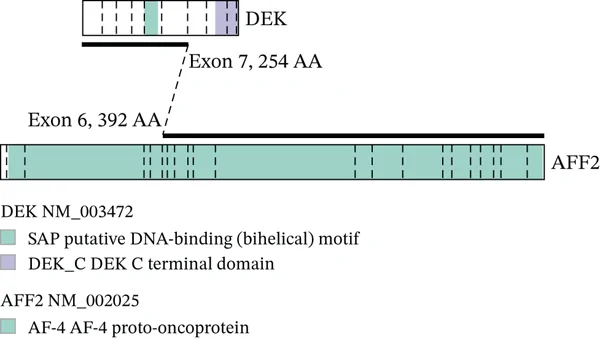
*DEK::AFF2* fusion schematic and protein domain organization. Figure was prepared using the ProteinPaint web application (Zhou et al., 2016) [8].

Targeted DNA sequencing of a 648‐gene panel, with concurrent RNA sequencing, was performed (Tempus Labs, Chicago, Illinois). RNA sequencing identified a *DEK::AFF2* fusion involving breakpoints at *DEK* exon 7 (chr6: 18249882) and *AFF2* exon 6 (chrX: 147924490), which is the defining molecular alteration of *DEK::AFF2* carcinoma (Figure 4). Initial DNA sequencing detected *TET2* p.Q913fs and p.Q913 ^∗^ mutations with a low variant allele fraction (VAF) of 8%–11.5%. However, *TET2* mutations were subsequently identified in peripheral blood as well, indicating that it represented tumor‐infiltrating clonal hematopoiesis (TI‐CH) rather than a tumor‐specific somatic event. No pathogenic alterations were identified in other commonly implicated sinonasal carcinoma‐associated genes, including *TP53*, *IDH2*, or *NUTM1*. PD‐L1 immunohistochemistry was positive, with a combined positive score (CPS) of 40. The tumor demonstrated a low tumor mutational burden (TMB) of 0.5 mutations/Mb and was microsatellite stable (MSS).

The patient underwent primary surgical resection including anterior craniofacial resection, right maxillectomy, right orbital exenteration, wide local excision of the facial skin, total rhinectomy, total bilateral ethmoidectomy, nasopharyngectomy, right parotidectomy, and right neck dissection. Histologic examination of the resection specimen showed metastatic carcinoma in 6 of 49 lymph nodes, with the largest metastatic focus measuring 2.0 cm (Figure 3). No extranodal extension was identified. The pathologic stage was pT4bN2b. The patient then completed adjuvant chemoradiation.

The clinical course was further complicated by aortic thrombosis and multiple cerebrovascular accidents. The patient died 8 months after diagnosis.

## 3. Discussion

This case represents the first reported *DEK::AFF2* fusion‐associated sinonasal carcinoma with both intracranial and orbital invasion, underscoring the aggressive clinical behavior of this rare entity.

The bland, papilloma‐like morphology contributes to the diagnostic challenge on limited biopsy and frozen sections, where the absence of overt cytologic atypia can obscure the malignant nature of the tumor. AFF2 immunohistochemistry may be used as a first‐line screening tool, but it is not widely available in all laboratories [9, 10]. *DEK* break‐apart FISH may be considered as an alternative confirmatory approach, though it does not identify the fusion partner. RNA sequencing remains the definitive diagnostic modality because it identifies the fusion partner, detects novel or alternative gene rearrangements, and simultaneously evaluates the broad molecular differential diagnosis of sinonasal carcinomas, including other fusion‐driven entities such as NUT carcinoma, SWI/SNF complex‐deficient sinonasal carcinoma, and adamantinoma‐like Ewing sarcoma. As the molecular landscape of sinonasal tumors continues to expand, combined RNA and DNA sequencing provides the most comprehensive approach to tumor classification and molecular characterization.

Despite their deceptively low‐grade histology, *DEK::AFF2* carcinomas demonstrate aggressive clinical behavior, including locally destructive growth, propensity for recurrence, and, as illustrated by the present case, capacity for extensive locoregional invasion involving the orbit and intracranial compartment. This discordance between histologic appearance and biologic behavior represents a critical clinical consideration, as underrecognition of the diagnosis may lead to inadequate initial surgical resection or delayed adjuvant therapy.

No standardized treatment protocol currently exists for *DEK::AFF2* carcinoma, reflecting the rarity of the entity. Management has generally followed principles applied to other sinonasal carcinomas. The Hart et al. multi‐institutional study of 30 *DEK*::*AFF2* fusion‐associated carcinomas treated the majority with surgery alone, a subset with combined‐modality therapy, and none with immunotherapy [6].

Gene fusions can generate immunogenic neoantigens capable of eliciting cytotoxic T‐cell responses and mediating responses to immune checkpoint inhibitors. Yang et al. described a patient with *DEK*::*AFF2* carcinoma who achieved a complete, durable response to anti–PD‐1 therapy despite a low TMB and sparse T‐cell infiltration [11]. However, PD‐L1 expression in *DEK*::*AFF2* carcinomas is variable. PD‐L1 was positive in approximately 40% of tumor cells by immunohistochemistry in a *DEK*::*AFF2* fusion‐associated carcinoma of the middle/external ear [12]. Trinquet et al. reported a broad range across cases (CPS 0–80; median 5) [5]. Clinical outcomes have mirrored this heterogeneity. Of the two patients treated with checkpoint inhibitors by Rooper et al., one patient initially had an excellent response to multimodality therapy despite metastatic disease but ultimately experienced recurrence and died 29 months after diagnosis, whereas the other showed no response to checkpoint inhibitors for lung metastases [3]. Najjar et al. reported a treatment‐resistant *DEK*::*AFF2* sinonasal squamous cell carcinoma that recurred repeatedly despite accurate molecular diagnosis, wide‐margin surgery, radiotherapy, and pembrolizumab‐based chemoimmunotherapy [13]. Taken together, these findings suggest that although responses vary, immune checkpoint inhibition can produce meaningful and even durable benefit in a subset of patients with *DEK*::*AFF2* carcinoma, supporting its consideration as part of a personalized, multimodal treatment strategy. PD‐L1 expression in the present case would have supported consideration of immune checkpoint inhibitor therapy. Further investigation into optimal therapeutic strategies will require collaborative multi‐institutional efforts given the rarity of this tumor.

Although *DEK*::*AFF2* carcinoma was initially defined as a nonkeratinizing squamous cell carcinoma, an expanding subset of reported cases has demonstrated glandular differentiation. Kuo et al., in validating the *AFF2* C‐terminus immunostain, encountered one of 17 molecularly confirmed *DEK*::*AFF2* carcinomas that showed glandular differentiation [10]. The overlap between *DEK*::*AFF2* carcinoma and sinonasal adenosquamous carcinoma is highlighted by Holliday et al., who identified a *DEK*::*AFF2* fusion in one of 11 sinonasal adenosquamous carcinomas, underscoring that this fusion can rarely drive tumors previously classified by their adenosquamous morphology and that such cases must be distinguished from HPV‐driven and *MAML2*‐rearranged mimics [14]. In their 15‐case cohort, Trinquet et al. identified one case with an unusual biphasic morphology comprising glandular formations lined by cuboidal luminal cells [5]. Zhai et al. found that more than 70% of 50 *DEK*::*AFF2* carcinomas coexpressed squamous and glandular immunohistochemical markers, with 6% displaying mucoepidermoid carcinoma‐like morphology and 4% an ameloblastoma‐like appearance [15]. In our case, the brain‐invasive foci demonstrated tumor cells in sheets and nests floating within pools of mucin, a pattern focally reminiscent of mucinous adenocarcinoma. These findings broaden the recognized histologic spectrum of this entity.

Recent work has further expanded the spectrum of *AFF2*‐rearranged carcinomas, with novel fusion partners including *H3-3A*, *EWSR1*, *CHD4*, and *NUCKS1* identified in sinonasal and parotid tumors, suggesting that *AFF2*‐rearranged carcinomas may form a broader family of molecularly related neoplasms [16]. One *EWSR1*::*AFF2* tumor exhibited high‐grade morphology with focal glandular areas showing mucin production and focal neuroendocrine marker expression, alongside nonglandular areas showing small round blue cell morphology with a high nuclear‐to‐cytoplasmic ratio, reinforcing the concept that *AFF2*‐rearranged carcinomas constitute a morphologically and immunophenotypically diverse spectrum [16].

A notable ancillary finding in this case was the identification of *TET2* mutations, which, through paired tumor‐blood sequencing, were determined to represent TI‐CH rather than somatic tumor‐associated events. TI‐CH is increasingly recognized as a confounding factor in tumor genomic profiling, as mutations arising in hematopoietic cells, particularly in genes such as *TET2*, *DNMT3A*, and *ASXL1*, may be detected in tumor specimens because of infiltrating immune cells [17]. Without paired germline or blood sequencing, such variants may be erroneously attributed to the tumor, potentially leading to misinterpretation of the tumor′s mutational landscape and inappropriate therapeutic decisions. This finding highlights the importance of incorporating paired tumor‐normal sequencing into the molecular characterization of rare tumors.

## 4. Conclusion

This case underscores the diagnostic challenges posed by the bland morphology of *DEK::AFF2* fusion‐associated carcinoma, the importance of molecular profiling in sinonasal neoplasms, and the emerging clinical relevance of TI‐CH in solid tumors.

## Funding

No funding was received for this manuscript.

## Ethics Statement

This study was conducted in accordance with institutional IRB requirements (H‐57403).

## Consent

Informed consent could not be obtained because the patient was deceased. No identifiable patient information is included in this report.

## Conflicts of Interest

The authors declare no conflicts of interest.

## Data Availability

The data that support the findings of this study are available on request from the corresponding author. The data are not publicly available due to privacy or ethical restrictions.

## References

[1] Skálová A. , Agaimy A. , Bradova M. , Poorten V. V. , Hanna E. , Guntinas-Lichius O. , Franchi A. , Hellquist H. , Simpson R. H. W. , Lopéz F. , Nuyts S. , Chiesa-Estomba C. , Ng S. P. , Homma A. , Teng Y. , Leivo I. , and Ferlito A. , Molecularly Defined Sinonasal Malignancies: An Overview With Focus on the Current WHO Classification and Recently Described Provisional Entities, Virchows Archiv. (2024) 484, no. 6, 885–900, 10.1007/s00428-024-03775-y, 38491228. 38491228 PMC11186917

[2] Todorovic E. , Truong T. , Eskander A. , Lin V. , Swanson D. , Dickson B. C. , and Weinreb I. , Middle Ear and Temporal Bone Nonkeratinizing Squamous Cell Carcinomas With DEK-AFF2 Fusion: An Emerging Entity, American Journal of Surgical Pathology. (2020) 44, no. 9, 1244–1250, 10.1097/PAS.0000000000001498, 32366754. 32366754

[3] Rooper L. M. , Agaimy A. , Dickson B. C. , Dueber J. C. , Eberhart C. G. , Gagan J. , Hartmann A. , Khararjian A. , London N. R. , MacMillan C. M. , Palsgrove D. N. , Nix J. S. , Sandison A. , Stoehr R. , Truong T. , Weinreb I. , and Bishop J. A. , DEK-AFF2 Carcinoma of the Sinonasal Region and Skull Base: Detailed Clinicopathologic Characterization of a Distinctive Entity, American Journal of Surgical Pathology. (2021) 45, no. 12, 1682–1693, 10.1097/PAS.0000000000001741, 34049316. 34049316

[4] Kuo Y. J. , Lewis J. S. , Zhai C. , Chen Y. A. , Chernock R. D. , Hsieh M. S. , Lan M. Y. , Lee C. K. , Weinreb I. , and Hang J. F. , DEK-AFF2 Fusion-Associated Papillary Squamous Cell Carcinoma of the Sinonasal Tract: Clinicopathologic Characterization of Seven Cases With Deceptively Bland Morphology, Modern Pathology. (2021) 34, no. 10, 1820–1830, 10.1038/s41379-021-00846-2, 34108636. 34108636

[5] Trinquet A. , Laé M. , Lépine C. , Lanic M. D. , Lacheretz-Szablewski V. , Shaar Chneker C. , Goujon J. M. , Favier V. , and Costes-Martineau V. , Sinonasal Squamous Cell Carcinoma With DEK::AFF2 Rearrangement: An Aggressive Cancer With Bland Morphology, American Journal of Surgical Pathology. (2024) 48, no. 11, 1408–1416, 10.1097/PAS.0000000000002281, 39132684. 39132684

[6] Hart S. A. , Hang J. F. , Chernock R. D. , Mikula M. W. , Rooper L. , Amin S. E. , Saluja K. , Bishop J. A. , Chen Y. H. , Cipriani N. A. , David S. N. , Dupont W. D. , Plummer W. D. , Ferrer K. T. , Geromes A. , Hsieh M. S. , Hernandez-Prera J. C. , Kuo Y. J. , Sasaki E. , Shi Q. , Truong T. , Velez Torres J. M. , and Lewis JS Jr , DEK::AFF2 Fusion Sinonasal and Skull Base Nonkeratinizing Squamous Cell Carcinoma: A Clinical Outcome Study Compared With Conventional Sinonasal Squamous Cell Carcinoma, American Journal of Surgical Pathology. (2025) 49, no. 2, 130–137, 10.1097/PAS.0000000000002335, 39719672. 39719672

[7] Knebel M. , Agaimy A. , Kühn J. P. , Körner S. , Braun F. , Brust L. , Flockerzi V. , Wemmert S. , Balensiefer B. , Schick B. , Yilmaz U. , Zaito M. , Bozzato A. , and Linxweiler M. , Case Report: “DEK::AFF2 Fusion Associated Sinonasal Carcinomas: A Novel Oncogenic Driver and Emerging Therapeutic Strategies”, Frontiers in Immunology. (2025) 16, 1611790, 10.3389/fimmu.2025.1611790, 40688086. 40688086 PMC12271193

[8] Zhou X. , Edmonson M. N. , Wilkinson M. R. , Patel A. , Wu G. , Liu Y. , Li Y. , Zhang Z. , Rusch M. C. , Parker M. , Becksfort J. , Downing J. R. , and Zhang J. , Exploring Genomic Alteration in Pediatric Cancer Using ProteinPaint, Nature Genetics. (2016) 48, no. 1, 4–6, 10.1038/ng.3466, 26711108. 26711108 PMC4892362

[9] Ng J. K. M. , Chow C. , Tang C. Y. , Chan A. Z. , Li J. J. X. , and Chan A. B. W. , Prevalence and Interpretation of AFF Immunostain of DEK::AFF2 Fusion-Associated Papillary Squamous Cell Carcinoma in a Retrospective Cohort of Recurrent Sinonasal Papillomas, Virchows Archiv. (2024) 484, no. 1, 119–125, 10.1007/s00428-023-03717-0, 38063896. 38063896

[10] Kuo Y. J. , Lewis J. S. , Truong T. , Yeh Y. C. , Chernock R. D. , Zhai C. , Chen Y. A. , Hongo T. , Lee C. K. , Shi Q. , Velez Torres J. M. , Geromes A. B. , Chu Y. H. , Hsieh M. S. , Yamamoto H. , Weinreb I. , and Hang J. F. , Nuclear Expression of AFF2 C-Terminus is a Sensitive and Specific Ancillary Marker for DEK::AFF2 Carcinoma of the Sinonasal Tract, Modern Pathology. (2022) 35, no. 11, 1587–1595, 10.1038/s41379-022-01117-4, 35701667. 35701667

[11] Yang W. , Lee K. W. , Srivastava R. M. , Kuo F. , Krishna C. , Chowell D. , Makarov V. , Hoen D. , Dalin M. G. , Wexler L. , Ghossein R. , Katabi N. , Nadeem Z. , Cohen M. A. , Tian S. K. , Robine N. , Arora K. , Geiger H. , Agius P. , Bouvier N. , Huberman K. , Vanness K. , Havel J. J. , Sims J. S. , Samstein R. M. , Mandal R. , Tepe J. , Ganly I. , Ho A. L. , Riaz N. , Wong R. J. , Shukla N. , Chan T. A. , and Morris L. G. T. , Immunogenic Neoantigens Derived From Gene Fusions Stimulate T Cell Responses, Nature Medicine. (2019) 25, no. 5, 767–775, 10.1038/s41591-019-0434-2, 31011208. PMC6558662 31011208

[12] Sun Y. W. , Zhou Y. , Liu X. Y. , and Shen D. H. , DEK::AFF2 Fusion-Associated Middle Ear Non-Keratinizing Squamous Cell Carcinoma: A Case Report, World Journal of Clinical Oncology. (2024) 15, no. 12, 1501–1506, 10.5306/wjco.v15.i12.1501, 39720645. 39720645 PMC11514374

[13] Najjar W. , Stemme R. , Mady L. J. , Seiwert T. Y. , Quon H. , Rooper L. M. , and Rowan N. R. , Treatment-Resistant DEK::AFF2 Sinonasal Squamous Cell Carcinoma: A Case Report and Review of Literature, Acta Oto-Laryngologica Case Reports. (2025) 10, no. 1, 186–191, 10.1080/23772484.2025.2558952.

[14] Holliday D. , Mehrad M. , Ely K. A. , Tong F. , Wang X. , Hang J. F. , Kuo Y. J. , Velez-Torres J. M. , Lott-Limbach A. , and Lewis J. S. , Sinonasal Adenosquamous Carcinoma–Morphology and Genetic Drivers Including Low- and High-Risk Human Papillomavirus mRNA, DEK::AFF2 Fusion, and MAML2 Rearrangement, Head and Neck Pathology. (2023) 17, no. 2, 487–497, 10.1007/s12105-023-01538-w, 36849671. 36849671 PMC10293130

[15] Zhai C. , Liu H. , Zhang J. , Wang S. , and Lin L. , DEK::AFF2 Fusion Sinonasal Tract and Skull Base Nonkeratinizing Squamous Cell Carcinoma: An Expanded Single-Center Series of 50 Cases Extending the Clinicopathologic Spectrum, American Journal of Surgical Pathology. (2026) 10.1097/PAS.0000000000002590, 42535378. 42535378

[16] Breimer G. E. , Hyrcza M. D. , Hahn E. , Prendergast S. C. , Smith S. M. , Chambers A. , Todorovic E. , Palsgrove D. , Miller D. L. , Heinhuis R. J. , Rijken J. A. , Kester L. A. , Wei C. , Weinreb I. , and Bishop J. A. , Expanding the Spectrum of AFF2 Carcinoma: Clinical, Morphological, Immunohistochemical, and Molecular Characteristics of Five Cases Harboring Alternate Fusions, Virchows Archiv. (2026) 488, no. 2, 345–354, 10.1007/s00428-025-04140-3, 40494991. 40494991 PMC12916527

[17] Pich O. , Bernard E. , Zagorulya M. , Rowan A. , Pospori C. , Slama R. , Huerga Encabo H. , O’Sullivan J. , Papazoglou D. , Anastasiou P. , Iliakis C. S. , Clark S. A. , Dijkstra K. K. , Barbè V. , Bailey C. , Stonestrom A. J. , Enfield K. S. S. , Green M. , Brierley C. K. , Magness A. , Pearce D. R. , Hynds R. E. , Zaidi R. , Rane J. K. , Álvarez-Prado Á. F. , Thol K. , Scott R. , Bola S. K. , Hoxha E. , Harris S. K. , Peggs K. S. , Quezada S. A. , Hackshaw A. , Zaccaria S. , Joyce J. A. , Malanchi I. , Berger M. F. , Jamal-Hanjani M. , Wack A. , Downward J. , Grey W. , Lo Celso C. , Grönroos E. , Rudin C. M. , Mead A. J. , Bonnet D. , Papaemmanuil E. , and Swanton C. , Tumor-Infiltrating Clonal Hematopoiesis, New England Journal of Medicine. (2025) 392, no. 16, 1594–1608, 10.1056/NEJMoa2413361, 40267425. 40267425 PMC12021423

